# Sole and Combined Application of Biodigestate, N, P, and K Fertilizers: Impacts on Soil Chemical Properties and Maize Performance

**DOI:** 10.1155/2024/6685906

**Published:** 2024-02-20

**Authors:** Aruna Olasekan Adekiya, Olufunmilayo Titilayo Ande, Samuel Olatunde Dahunsi, Joshua Ogunwole

**Affiliations:** ^1^Agriculture Programme, College of Agriculture, Engineering and Science, Bowen University, Iwo, Nigeria; ^2^Microbiology Programme, College of Agriculture, Engineering and Science, Bowen University, Iwo, Osun, Nigeria

## Abstract

The fertilizing effects of biodigestate produced from biogas plants on crop and soil productivity are very scarce. Hence, a field study was conducted in 2022 at the Teaching and Research Farm of Bowen University, Iwo, Osun State, Nigeria. The study evaluated the effects of biodigestate fertilizer, applied alone or in combination with urea, single superphosphate, or muriate of potash fertilizers at low (N_1_, K_1_, and P_1_) and high (N_2_, P_2_, and K_2_) rates on soil chemical properties, growth, and yield of maize (*Zea mays* (L.)). The treatments were biodigestate alone (D), D + N fertilizer (urea) at 60 kg·ha^−1^ (DN_1_), D + N at 120 kg·ha^−1^ (DN_2_), D + P fertilizer (single superphosphate) at 30 kg·ha^−1^ (DP_1_), D + P at 60 kg·ha^−1^ (DP_2_), D + K fertilizer (muriate of potash) at 30 kg·ha^−1^ (DK_1_), D + K 60 kg·ha^−1^ (DK_2_), D + N_1_ + P_1_ + K_1_ (DN_1_P_1_K_1_), D + N_2_ + P_2_ + K_2_ (DN_2_P_2_K_2_) (10), and control. The 10 treatments were arranged in a randomized complete block design and replicated three times. Results showed that both low and high rates of fertilizer application improved soil chemical properties, growth parameters, and yield of maize compared with the control. High fertilizer rates (N_2_, P_2_, and K_2_) significantly enhanced soil chemical properties and growth parameters, but lower rates (N_1_, P_1_, and K_1_) resulted in higher maize yield. DN_1_ fertilizer significantly increased maize yield compared with DN_2_, DP_1_, DP_2_, DK_1_, and DK_2_. Overall, the treatment of DN_1_P_1_K_1_ demonstrated the highest grain yield, likely due to optimal nutrient supply from N, P, and K fertilizers, along with an improved soil environment facilitated by the biodigestate. The study recommends a balanced and sustainable fertilizer application strategy of 60 kg·N·ha^−1^, 30 kg·P_2_O_5_·ha^−1^, and 30 kg·K·ha^−1^ with 2500 L·ha^−1^ of biodigestate to enhance maize production while minimizing cost and environmental impact. However, for those aiming for maize fodder production, a higher fertilizer rate of 120 kg·N·ha^−1^, 60 kg·P_2_O_5_·ha^−1^, and 60 kg·K·ha^−1^ with 2500 L·ha^−1^ of biodigestate is advised.

## 1. Introduction

The world population is estimated to reach 9 billion by 2050 [1, 2]; therefore, there will be more food to feed the continuously growing population, especially in developing countries like Nigeria. Food stands as a paramount essential for humanity. To achieve the millennium development goal in food production, particularly in Nigeria, an abundant supply of food must be ensured. The potential threat to food security due to dwindling soil fertility necessitates urgent attention. Several limitations significantly hinder the viability of agricultural sustainability in tropical soils. These include high bulk density, a diminished capacity to retain water and nutrients, and the soil's rapid mineralization of organic matter. As a result, the majority of soils are not conducive to achieving high crop yields due to their lightweight texture and inadequate nutrient reserves [3]. Within tropical regions, sandy soils exhibit low levels of organic matter and essential nutrients such as nitrogen, phosphorus, potassium, calcium, and magnesium.

In addition, they possess a low cation exchange capacity (CEC) and have a limited capacity to store and provide soil moisture [4]. For the foregoing reasons, farmers abandon the land for another after cultivating it for 2-3 years; however, due to the scarcity of land, there is a need to increase the yield per unit area of the existing cultivated lands. Enhancing soil productivity and increasing yield per unit area can be achieved through incorporating external inputs, such as organic and chemical fertilizers [5].

In this pursuit, the judicious application of fertilizers, including nitrogen (N), phosphorus (P), and potassium (K), has been a longstanding agricultural practice [6]. These essential nutrients play critical roles in various physiological and biochemical processes vital for plant growth and development. However, conventional fertilizer use has raised concerns related to environmental sustainability, soil health, and the associated economic costs [5].

To address these concerns, there has been a burgeoning interest in exploring alternative fertilization approaches that mitigate environmental impact and optimize nutrient utilization efficiency. One promising avenue is the use of biodigestate, organic matter derived from the anaerobic digestion of agricultural residues and organic waste, into the fertilization regimen. Just like organic manures, biodigestate not only provides essential nutrients but also enhances soil structure, microbial activity, and overall soil health [7]. The agricultural and horticultural sectors show significant promise for the utilization of biodigestate. It is commonly employed as a soil conditioner and organic fertilizer in these fields due to its abundance of nutrients and beneficial microorganisms for soil health [8, 9]. The application of biodigestates is particularly beneficial for enhancing and modifying soil structures, improving soil nutrient levels, and fostering a variety of beneficial microorganisms with specific functions, especially in soils that are marginal or depleted in nutrients when used as organic fertilizers [10]. Notably, digestate serves as a slow-release fertilizer, supplying essential nutrients like nitrogen, phosphorus, and potassium (NPK), along with other crucial plant macronutrients necessary for the growth, health, and overall well-being of crop plants. Importantly, these positive effects are achieved without causing any harm to the soil [11, 12]. Currently, numerous studies are dedicated to exploring the impact of digestates on crop and vegetable yields as well as quality [13, 14].

Therefore, when combined with conventional N, P, and K fertilizers, biodigestate has the potential to synergistically impact soil chemical properties and subsequently influence crop performance.

Maize, scientifically known as *Zea mays* (L.), stands out as a highly efficient and industrially significant cereal crop on a global scale. It holds the esteemed position of being the third most prominent cereal crop worldwide, following wheat and rice. Moreover, maize holds immense importance as a staple food in numerous countries. Its grains typically contain approximately 13% moisture, 10% crude protein, and impressive 70.3% carbohydrate content [15]. As highlighted by Nweke [16], maize constitutes a significant portion, approximately 43%, of the caloric intake in an average Nigerian diet. The fresh maize grains offer culinary versatility; they can be roasted or cooked for immediate consumption. Alternatively, after undergoing the milling and boiling processes, the grains can be transformed into porridge. Maize shares a characteristic trait with other cereal crops; it is a nutrient-demanding plant, necessitating a rich nutrient supply for optimal growth and heightened productivity.

Attaining optimal maize yields necessitates a well-rounded and sufficient provision of nutrients, given that the reduction in soil fertility poses a significant hurdle for maize cultivation [17]. Maize, like other cereal crops, is a heavy feeder and needs a high amount of nutrients for increased productivity [5]. The use of chemical fertilizer has been reported [5, 18] to increase maize yields, but in Nigeria, its use is limited by high cost and scarcity during the time of its need (planting season) [18]. Because of these, the use of organic fertilizers such as biodigestate was found to be useful in increasing crop production. Also, previous studies have shown that the appropriate use of biodigestate can increase soil nutrients, enhance crop nutritional quality, reduce greenhouse gas emissions, decrease crop disease, and have other benefits [19, 20]. However, biodigestate like other organic manures is limited by the slow-release nature of its nutrient [21], especially at the initial growth stage of maize. Therefore, to avert this problem, there is strong advocacy for integrating organic and inorganic fertilizers [22, 23].

Several studies have been conducted on the integrated effect of organic and inorganic fertilizers on maize performance [24–26]. It is important to conduct research to explore the appropriate N, P, and K levels with supplemental incorporation of biodigestate (organic fertilizer) for achieving optimum soil chemical properties and crop yield, as little overapplication of any of the fertilizers could be economical due to its cost.

In addition, this work investigates the sole and combined application of biodigestate, N, P, and K fertilizers, and their effects on soil chemical properties and maize performance. The study seeks to contribute valuable insights that can inform sustainable agricultural practices, reduce the cost of fertilization, and promote optimal nutrient utilization and crop yields while safeguarding environmental and economic sustainability.

## 2. Materials and Methods

### 2.1. Site Description and Treatments

A field study was conducted in July 2022 at the Teaching and Research Farm of Bowen University, located in Iwo, Osun State, Nigeria (coordinates 7.6236°N, 4.1890°E, altitude 312 m above sea level). The soil, identified as sandy loam, formed from fine-grained granite gneiss and schist and is correlated with the Egbeda series (plinthic) as reported by Smyth and Montgomery [27] in their study of soils in Southwestern Nigeria. The soil in this area is also similar to soils described as Plinthic Kandiudalf by Ajiboye et al. [28], following the classification of Soil Survey Staff [29]. The soil was formed in residuum, with an abrupt increase in clay content in the subsoil, and also possessed plinthic properties in the horizon within the first 150 cm of the mineral horizons. The pedogenesis of such soils formed from the basement complex in residuum has been described by Ande and Senjobi [30]. The region experiences a bimodal pattern of rainfall, averaging around 1300 mm annually, and maintains an average temperature of 32°C. The climate is classified as subhumid.

The experiment consisted of 10 treatments and are listed as follows: sole application of biodigestate at 2500 L·ha^−1^ (D), application of biodigestate at 2500 L·ha^−1^ with sole application of N fertilizer (urea) at 60 kg·ha^−1^ (DN_1_) and 120 kg·ha^−1^ (DN_2_) [18], application of biodigestate at 2500 L·ha^−1^ with sole application of P fertilizer (single superphosphate) at 30 kg·ha^−1^ (DP_1_) and 60 kg·ha^−1^ (DP_2_) [31], application of biodigest at 2500 L·ha^−1^ with sole application of K fertilizer (muriate of potash) at 30 kg·ha^−1^ (DK_1_) and 60 kg·ha^−1^ (DK_2_) [32], application of biodigestate at 2500 L·ha^−1^ + urea fertilizer at 60 kg·ha^−1^ + single superphosphate at 30 kg·ha^−1^ + muriate of potash at 30 kg·ha^−1^ (DN_1_P_1_K_1_), application of biodigestate at 2500 L·ha^−1^ + urea fertilizer at 120 kg·ha^−1^ + single Superphosphate at 60 kg·ha^−1^ + muriate of potash at 60 kg·ha^−1^ (DN_2_P_2_K_2_), and no application of any soil amendment (control) (D_0_N_0_P_0_K_0_). The treatments were arranged in a randomized complete block design and replicated three times.

### 2.2. Land Preparation, Field Layout, Application of Biodigestate, and Sowing of Maize Seeds

The designated experimental area was ploughed and harrowed before delineating the field layout promptly post-harrowing using ropes, pegs, and tape. Each experimental plot measured 12 × 3 meters and was subjected to a specific treatment, replicated three times. The plots were spaced at intervals of 0.5 meters, while blocks were separated by 1 meter. The biodigestate used in this study was produced from the anaerobic codigestion of poultry droppings and piggery dung. A 20,000 l capacity digester currently used for the digestion of animal wastes accrued in the Bowen University Farm was used. It is a continuous digester with constant supply of poultry droppings and piggery dung, while the inoculum was from cow dung. In practice, the wastes are continuously digested in the digestion chamber, while the biodigestate slurry which is produced as a by-product after methane generation is channeled into a 1,000 l capacity holding chamber for collection. After collection of the slurry, it was analysed for various important parameters prior to the soil application. The biodigestate was incorporated into the soils almost simultaneously with planting after the experiment layout on the field at the rate of 2500 L·ha^−1^, which is equivalent to 9 L plot^−1^. A hand-held hoe was used to incorporate the biodigestate into the soil to a depth of approximately 20 cm. Hybrid maize from Seed Co, which is high yielding, was sown on the 8th of August 2022 at a depth of 2-3 cm. This variety is medium maturing and embedded with vitamin A. It can tolerate crowding and high population. Two seeds were planted in each hole with a spacing of 75 cm × 25 cm. Two weeks from the sowing date, the seedlings were thinned down to one plant per stand, resulting in a total of 192 plants per plot, approximately amounting to 53,333 plants per hectare. P fertilizer (single superphosphate 20% P_2_O_5_) was applied at the rate of 30 kg·ha^−1^ and 60 kg·ha^−1^, respectively, for P_1_ and P_2_ treatments at sowing, while K fertilizer (muriate of potash—60% K) was applied at 2 weeks after sowing (WAS) of maize. K fertilizer was applied at the rates of 30 kg·ha^−1^ and 60 kg·ha^−1^, respectively, for K_1_ and K_2_ treatments. Urea fertilizer (46% N) was split-applied; the first dose of 30 kg·ha^−1^ (N_1_ treatments) and 60 kg·ha^−1^ (N_2_ treatments) was made 3 WAS, while the remaining dose was applied 6 WAS. Urea and muriate of potash fertilizers were administered by side placement, positioned approximately 8–10 cm away from the seeds during the sowing process and at the base of the plant following germination at a depth of about 3 cm. Weeds were controlled with Paraforce (paraquat dichloride), a preplanting herbicide at a rate of 1.00 kg ai/ha and atrazine, a selective and systemic herbicide containing 80% atrazine WP at a rate of 1.5 kg ai/ha. Fall armyworm incidence was treated with caterpillar force (emamectin benzoate 5% WDG), a nonsystemic insecticide at a rate of 30 mL per 10 L of water two WAS and repeated at 4 WAS and the 6 WAS for effective control. Five plants were tagged from each plot for data collection.

### 2.3. Soil and Biodigestate Analysis

Surface soil samples were randomly taken from the experimental field at a depth of 0–15 cm for subsequent physical and chemical analyses before commencing the experiment. The collected soil samples were air-dried, sieved through a 2-mm sieve, and preserved for analysis. The sand, silt, and clay contents were determined using the hydrometer method [33]. Soil pH was measured using a pH meter with a 1 : 2.5 soil/water ratio. Total nitrogen content was determined using the micro-Kjeldahl method [34], available phosphorus was assessed using the Bray 1 method [35], and calcium (Ca) and magnesium (Mg) were analysed using atomic absorption spectrophotometry (AAS). Potassium (K) and sodium (Na) levels were measured using flame emission photometry, following the procedure outlined by the Association of Official Analytical Chemists [36]. Organic carbon content was determined using the dichromate wet oxidation method according to Walkey and Black, as detailed by Nelson and Sommers [37]. Upon completion of the experiment, soil samples were once again collected randomly from five distinct locations within each plot from 0 to 15 cm soil depth. These samples were combined to create composite soil samples on a plot basis and subjected to the same abovementioned chemical analysis procedures.

A sample of the biodigestate was also collected for analysis of the following parameters: total nitrogen, available phosphorus, and exchangeable potassium, calcium, and magnesium, as described by Tel and Hagarty [38].

### 2.4. Determination of Growth and Yield Parameters

In each plot, ten maize plants were designated for data collection purposes. Growth-related information was gathered at the tasseling stage of the maize plants. The specified parameters encompassed plant height, leaf count, stem circumference, and leaf length. Plant height was measured with a tape measure, extending from the plant's base to its tassel. Leaf count was determined by simple enumeration. The stem circumference was measured using a Vernier caliper. Leaf length was assessed using a tape measured from the sheath to the tip of the leaves. The maize plants were allowed to undergo the drying process before the harvest. At the maize harvest (90 days after sowing), parameters pertaining to yield were recorded, including biomass weight, ear weight, cob weight, and shelled grain weight.

Biomass weight was measured by cutting and weighing the entire maize plant (including stalks, leaves, ears, and cobs). Ear weight was deduced by detaching the ears from the harvested plants and weighing them. Shelled grain weight was determined by shelling the kernels from the cobs and weighing them.

### 2.5. Data Analysis

The data collected for growth and yield parameters underwent statistical analysis through Analysis of Variance (ANOVA) using the Statistical Analysis System (SAS) [39], version 9.4. Mean values were then distinguished through Tukey pairwise comparisons at a significance level of *p* < 0.05.

## 3. Results

### 3.1. Initial Soil Characteristics of the Site Used

The initial soil characteristics of the site used for the experiment in 2022 are presented in Table 1. The experimental site was slightly alkaline and sandy loam in texture. The site was low in organic matter (OM), N, and P and slightly acidic. The exchangeable bases K, Ca, and Mg were adequate according to the critical levels of 3.0% OM, 0.2.0% N, 10.0 mg·kg^−1^ P, 0.16–0.20 cmol·kg^−1^ K, 2.0 cmol·kg^−1^ Ca, and 0.40 cmol·kg^−1^ Mg recommended for crop production in the agroecological zone in Nigeria [40]. Chemical analysis of biodigest used (Table 2) indicates that it contains nutrient elements (N, P, K, Ca, and Mg) required for the growth of a cereal crop such as maize.

### 3.2. Effects of Biodigestate, N, P, and K Fertilizers on Soil Chemical Characteristics

The results of the effects of biodigestate, N, P, and K fertilizers on soil chemical characteristics are presented in Table 3. Application of biodigestate alone (D) or in combination with N, P, and K fertilizers at any rate increased the pH, OC, N, P, K, Ca, Na, and Mg content of the soil relative to the control. The high rate of fertilizer (N_2_, P_2_, and K_2_) increased soil chemical properties relative to low rates (N_1_, K_1_, and P_1_). DN_2_P_2_K_2_ increased soil chemical properties relative to DN_1_P_1_K_1_. The integration of D with either N, P, or K fertilizer or their combination N + P + K (DN_1_P_1_K_1_ and DN_2_P_2_K_2_) increased soil chemical properties relative to D alone. In relative terms, among the combination of D with N, P, and K, DN_2_ increased soil chemical properties the most.

### 3.3. Effects of Biodigestate, N, P, and K Fertilizers on the Growth and Yield of Maize

Tables 4 and 5, respectively, show the result of the effect of biodigestate, N, P, and K fertilizers on some growth (plant height, number of leaves, leaf length, and stem girth) and yield (biomass weight, ear weight, cob weight, and grain weight) parameters of maize. Application of biodigestate alone (D) or in combination with N, P, and K fertilizers at any rate increased the growth and yield parameters of maize relative to the control. There were no significant differences between biodigestate alone (D) or in combination with N, P, and K fertilizers and the control treatment for a number of leaves/plants (Table 4). The high rate of fertilizer (N_2_, P_2_, and K_2_) increased the growth parameters of maize relative to low rates (N_1_, K_1_, and P_1_). DN_2_P_2_K_2_ increased maize growth compared with DN_1_P_1_K_1_ (Table 4). The integration of D with either N, P, or K fertilizer or their combination N + P + K (DN_1_P_1_K_1_ and DN_2_P_2_K_2_) increased maize growth relative to D alone. The low rate of fertilizer (N_1_, P_1_, and K_1_) increased the yield parameters of maize relative to high rates (N_2_, K_2_, and P_2_). DN_1_P_1_K_1_ increased maize yield relative to DN_2_P_2_K_2_ (Table 5). In relative terms, among the combinations of D with N, P, and K, DN_1_ increased the yield of maize the most. Relative to DN_2_, DP_1_, DP_2_, DK_1_, and DK_2_, DN_1_ increased grain weight of maize by 65.4, 56.0, 147.0, 8.98, and 24.1%, respectively. DN_1_P_1_K_1_ increased maize yield compared with DN_2_P_2_K_2_. Overall, DN_1_P_1_K_1_ has the highest grain yield.

## 4. Discussion

The experimental site's soil exhibited low nutrient levels, particularly in terms of soil organic carbon (OC), nitrogen (N), and phosphorus (P). These soil conditions are typical of tropical regions. The inadequate levels of total N and available P before planting were attributed to the low organic matter in the soil.

Results (Table 1) show that the site was low in OC, N, P, K, Ca, and Mg. These conditions are the characteristics of Alfisols in southwest Nigeria [41, 42]. Adekiya et al. [43] and Agbede et al. [3] earlier found that tropical soils are low in soil fertility. The inadequate levels of total N and available P before planting were attributed to the low organic matter in the soil. Agbede et al. [3] also attributed the low N and P on preplanting soil to low organic matter.

The use of biodigestate alone (D) or in combination with N, P, and K fertilizers at various rates led to an improvement in soil characteristics, including an increase in pH, organic carbon (OC), nitrogen (N), phosphorus (P), potassium (K), calcium (Ca), sodium (Na), and magnesium (Mg) content compared to the control. This outcome was anticipated due to the initially low soil fertility, as indicated in (Table 1). Urea fertilizer, muriate of potash (potassium chloride), and single superphosphate are commonly used in agriculture to provide essential nutrients to plants and improve soil fertility. Each of these fertilizers has specific effects on soil properties and nutrient content, which can influence soil pH and nutrient levels relative to a control (soil without fertilizer). Biodigestate also contains nutrients (Table 2), and these nutrients are released into the soil and used by plants. The increase in soil content of basic macronutrients was also observed in other studies [44, 45], which can be attributed to their high content in the organic material used. In an experiment to assess the fertilizing effects of digestate on chemical and biological soil properties in a field experiment in eastern Portugal with two horticultural crops [46], in addition to N, digestate supplied significant amounts of P, Ca, K, and Mg and significantly increased soil Olsen P, mineral N, and organic C. It was reported that the application of biogas digestate significantly increased soil pH, content of organic carbon, total N, and available forms of P, K, and Mg [47, 48]. According to Smith et al. [49], biogas digestate has great potential to increase soil C sequestration.

It increased soil OC because the biodigestate was prepared from organic waste (cow dung). This result is not in line with the result of Muhammad [50] which reported that the application of bio-slurry in liquid and composted form brought no change in the organic matter content of the soil. The rise in soil organic matter and nutrient levels, stemming from the usage of urea, muriate of potash, and single superphosphate fertilizers, might be attributed to the accelerated and robust growth of maize plants following their application. In addition, the deposition of crop residues, particularly leaves, during plant maturity could have contributed organic matter to the soil. This collective input has led to modest augmentation in the soil's organic matter, as well as its nitrogen (N), phosphorus (P), potassium (K), calcium (Ca), and magnesium (Mg) content [22]. Debebe and Itana [51] also revealed in their study that bio-slurry increases organic carbon, phosphorus, and cation exchange capacity of soil. Alemneh [52] also reported an increase in soil chemical properties due to bio-slurry application.

The high rate of fertilizer (N_2_, P_2_, and K_2_) and DN_2_P_2_K_2_ increased soil chemical properties relative to low rates (N_1_, K_1_, and P_1_) and DN_1_P_1_K_1_ due to higher concentrations of nutrients in higher rates relative to lower rates.

The integration of D with either N, P, or K fertilizer or their combination N + P + K (DN_1_P_1_K_1_ and DN_2_P_2_K_2_) increased soil chemical properties relative to D alone. This was due to the high quantity of N, P, and K in urea, single superphosphate, and muriate of potash fertilizer, respectively, compared with D. Biodigestate (D) contains a diverse range of nutrients due to its organic origin, including nitrogen, phosphorus, potassium, and various micronutrients. Combining this with urea, muriate of potash, and single superphosphate, which are concentrated N, K, and P sources, offers a more comprehensive and balanced nutrient supply to the soil, enriching its chemical properties.

Biodigestate (D) increased the growth and yield of maize in this study compared with the control. This was due to the high concentration of nutrients in D and subsequent nutrient release. In a comparative experiment aiming to assess the impact of bio-slurry and synthetic fertilizer on soil characteristics, growth, and yield of white cabbage [51], it was observed that the application of bio-slurry led to an increase in cabbage yield compared to the control group.

The enhanced growth and yield of maize observed after the application of N, P, and K fertilizers can be attributed to the deficiency of vital nutrients in the soil at the experimental site, crucial for the growth and yield of maize. Ogunboye et al. [18] and Amali and Namo [53] reported an increase in maize yield due to urea fertilizer. Ahmad et al. [54] and Ademba et al. [55] reported an increase in maize yield due to phosphate fertilizer. Ul-Allah et al. [56] also recorded a significant increase in the yield of maize due to potassium fertilizer. The reason for these responses was that nitrogen present in urea fertilizer stimulates leaf growth and is essential for protein and chlorophyll formation. On the other hand, phosphorus in superphosphate aids in root development, energy transfer reactions, and cell division and multiplication. In addition, potassium supports stem development, cell division, and the formation and movement of carbohydrates from source to sink.

The integration of D with either N, P, or K fertilizer or their combination N + P + K (DN_1_P_1_K_1_ and DN_2_P_2_K_2_) increased maize growth relative to D alone. The elemental content of nitrogen (N), phosphorus (P), and potassium (K) in urea, muriate of potash, and single superphosphate fertilizers exceeded that found in biodigestate. However, upon addition to these fertilizers, biodigestate, being of organic origin, contributes organic matter to the soil. This organic matter enhances soil structure, augments water retention, and boosts the microbial activity. In addition, this organic matter serves as a gradual-release nutrient source, providing consistent nourishment to maize. The humus generated by the biodigestate aids in retaining nutrients released from swiftly mineralized chemical fertilizers within the rooting zone [57], preventing leaching commonly observed in tropical soils. Consequently, these fosters improved nutrient uptake efficiency and heightened yields compared to using biodigestate alone or employing no additives. The presence of biodigestate may have also aided in maintaining a higher amount of applied urea in the soil, either in its original form or as ammonium ions, for an extended duration. This, in turn, resulted in enhanced nitrogen uptake efficiency [58, 59]. Debebe and Itana [51] similarly noted a significant increase in cabbage yield when bio-slurry was combined with chemical fertilizer as opposed to using bio-slurry in isolation. These findings align with the research of Dinka et al. [60] and Naiji and Souri [61], which indicate that an integrated nutrient approach led to higher crop yields compared to relying solely on recommended inorganic or organic fertilizers. The authors further emphasized that such integration of organic and inorganic fertilizers has the potential to enhance soil productivity and quality over the long term.

The high rate of fertilizer (N_2_, P_2_ and K_2_) increased the growth parameters of maize relative to low rates (N_1_, K_1_, and P_1_), whereas higher yield was recorded at the low rate (N_1_, K_1_, and P_1_) relative to the high rate of fertilizer (N_2_, P_2_ and K_2_). This result could be because of higher nutrient concentrations at the (N_2_, P_2_ and K_2_) level (Table 3), and the maize plant might have a focus on vegetative development, such as leafy biomass, at the expense of reproductive structures such as ears and kernels, leading to lower yields. Conversely, a maize plant with lower growth (N_1_, K_1_, and P_1_) might allocate more resources towards reproductive structures, resulting in a higher yield despite limited vegetative growth. Brewbaker [62] found that as nutrient levels increase, the overall biomass yield of maize plants will continue to rise, primarily due to plant enlargement rather than an increase in grain yield. Before now, White [63] similarly noted that elevated levels of nitrogen may slightly enhance the maize yield, but the cost-effectiveness of this improvement is questionable. In addition, higher phosphorus rates did not elicit any noticeable response in yield in maize.

DN_1_ increased the yield of maize relative to DN_2_, DP_1_, DP_2_, DK_1_, and DK_2_. These yield responses to fertilization show that N deficiency was the most limiting condition in maize production in the area. Although, DN_1_ was able to be adjudged the best and surpassed DK_1_ probably due to the higher initial K content of the soil (Table 1). Apart from N, K is another important nutrient necessary for maize cultivation [64]. Nitrogen promotes the growth of leaves, stems, and overall vegetative parts of the maize plant. It is essential for lush, green foliage and robust plant structure, whereas K is important among other things in supporting the filling of maize kernels, leading to higher grain weight and improved quality. It is particularly important during the grain-filling stage. Under North American conditions, a maize crop producing 9.5 tonnes of grain per hectare can remove 191 kg N·ha^−1^, 89 kg P_2_O_5_·ha^−1^, and 235 kg K_2_O ha^−1^ [64].

Overall, DN_1_P_1_K_1_ has the highest grain yield, which could be a result of optimum nutrient supply from N, P, and K fertilizers and better physical soil environment created by the biodigestate.

## 5. Conclusion

Biodigestate (D) fertilizer applied alone or in combination with sole urea (N), single superphosphate (P), or muriate of potash (K) fertilizer or their combinations (N + P + K) at low (N_1_, K_1_, and P_1_) and high (N_2_, P_2_, and K_2_) rates improved soil chemical properties, growth, and yield of maize compared with the control. The integration of D with either N, P, or K fertilizer or their combination N + P + K (DN_1_P_1_K_1_ and DN_2_P_2_K_2_) increased soil chemical properties relative to D alone. In relative terms, among the combination of D with N, P, and K, DN_2_ increased soil chemical properties the most.

The high rate of fertilizer (N_2_, P_2_ and K_2_) increased soil chemical properties and growth parameters of maize relative to low rates (N_1_, K_1_, and P_1_), whereas higher yield was recorded at the low rate (N_1_, K_1_ and P_1_) relative to the high rate of fertilizer (N_2_, P_2_, and K_2_). These results could be because of higher nutrient concentrations at the (N_2_, P_2_, and K_2_) level which might have made the maize plant focus on vegetative development, such as leafy biomass, at the expense of reproductive structures such as ears and kernels, leading to lower yields. DN_1_ increased the yield of maize relative to DN_2_, DP_1_, DP_2_, DK_1_, and DK_2_. Overall, DN_1_P_1_K_1_ has the highest grain yield, which could be as a result of optimum nutrient supply from N, P, and K fertilizers and better physical soil environment created by the biodigestate. Therefore, to avoid waste of fertilizer due to cost and negative environmental effect excessive fertilization, the lower rate of N (60 kg N ha^−1^), P (30 kg P_2_O_5_ ha^−1^), and K 30 kg K ha^−1^ fertilizers with 2500 L·ha^−1^ of biodigestate is recommended for sustainable maize production; however, for those who want to use maize as fodder, a higher rate of N (120 kg N ha^−1^), P (60 kg P_2_O_5_ ha^−1^), and K 60 kg K ha^−1^ fertilizers with 2500 L·ha^−1^ of biodigestate is recommended.

## Figures and Tables

**Table 1:** Initial soil characteristics of the experimental site before maize sowing.

Property	Value
Sand (%)	68.2
Silt (%)	19.1
Clay (%)	12.7
Textural class	Sandy loam
Organic C (%)	1.70
pH (water)	6.33
N (%)	0.17
P (mg·kg^−1^)	5.07
K (cmol·kg^−1^)	0.25
Ca (cmol·kg^−1^)	4.47
Mg (cmol·kg^−1^)	2.46
Na (cmol·kg^−1^)	0.26

**Table 2:** Nutrient values of the biodigestate used.

Nutrient	Value
N (%)	1.50
P (%)	1.10
K (%)	1.00
Ca (%)	0.46
Mg (%)	1.01

**Table 3:** Chemical characteristics of the soil after maize harvest.

	pH (water)	Ca (cmol·kg^−1^)	Mg (cmol·kg^−1^)	Na (cmol·kg^−1^)	K (cmol·kg^−1^)	Org C (%)	N (%)	P (mg kg^−1^)
DN_1_	6.49ab	3.37e	2.87d	0.40b	0.16e	1.60d	0.20b	22.9c
DN_2_	6.56a	4.06b	3.11bc	0.50a	0.21d	1.95b	0.23a	24.2b
DP_1_	6.63a	3.38e	3.01c	0.46a	0.19d	1.65d	0.14d	25.6b
DP_2_	6.64a	3.66d	3.30b	0.50a	0.20d	1.77c	0.18c	27.7a
DK_1_	6.57a	3.11f	2.83d	0.48a	0.24c	1.73c	0.17c	22.6c
DK_2_	6.58a	4.01b	3.29b	0.51a	0.28b	1.88c	0.19b	23.9b
DN_1_P_1_K_1_	6.51a	3.80c	2.46d	0.43b	0.23c	1.80c	0.18c	24.3b
DN_2_P_2_K_2_	6.70a	4.51a	3.51a	0.51a	0.35a	2.22a	0.24a	28.3a
D only	6.45ab	2.66g	3.10c	0.51a	0.20d	2.07b	0.20b	22.7c
D_0_N_0_P_0_K_0_ (control)	5.99c	2.44h	2.02e	0.24c	0.14f	1.51e	0.12e	3.31d

Values followed by similar letters under the same column are not significantly different at *p*=0.05 according to Duncan's multiple range test.

**Table 4:** Growth parameter of maize after application treatments.

Treatment	Plant height	Average number of leaves/plants	Leaf length	Stem girth
DN_1_	137.3d	9.5a	73.1cd	6.1cd
DN_2_	145.3c	10a	76.3c	6.3cd
DP_1_	138.3d	9.8a	74.0cd	6.1cd
DP_2_	143.0c	10.3a	77.4c	6.3cd
DK_1_	146.8c	10a	74.2cd	7.6b
DK_2_	149.1c	11a	88.1a	8.0a
DN_1_P_1_K_1_	159.0b	11a	81.7b	8.0ab
DN_2_P_2_K_2_	174.7a	11.3a	88.7a	8.4a
D only	129.8e	9.3a	75.1cd	7.3b
D_0_N_0_P_0_K_0_ (control)	110.2f	9.2a	69.2e	5.7 d

Values followed by similar letters under the same column are not significantly different at *p*=0.05 according to Duncan's multiple range test.

**Table 5:** Yield parameter of maize after application treatments.

Treatment	Biomass weight (kg)	Ear weight (kg)	COB weight (kg)	Grain weight (t·ha^−1^)
DN_1_	9.29c	5.19	4.91	5.46c
DN_2_	6.02g	3.56	2.97	3.3g
DP_1_	6.79f	3.72	3.2	3.56f
DP_2_	4.43h	2.48	1.99	2.21h
DK_1_	8.86d	5.19	4.63	5.01d
DK_2_	7.89e	4.68	4.01	4.46e
DN_1_P_1_K_1_	13.0b	8.7	7.61	8.46a
DN_2_P_2_K_2_	13.6a	7.68	6.71	7.46b
D only	9.78c	4.82	3.86	4.29e
D_0_N_0_P_0_K_0_ (control)	4.03i	2.30	1.34	1.49i

Values followed by similar letters under the same column are not significantly different at *p*=0.05 according to Duncan's multiple range test.

## Data Availability

All data used are included within the article.
